# New Appliances and Things Medical

**Published:** 1911-03-11

**Authors:** 


### NEW APPLIANCES & THINGS MEDICAL.

### HORLICK'S MALTED MILK.

(Horlick's Malted Milk Co., Slough, Bucks.)

We have received new samples of this well-known dietetic preparation, which clearly show that the standard maintained by the makers is a very high one. Nothing is easier to prepare than Horlick's; no other milk preparation containing the same amount of soluble protein and carbohydrate is more palatable when prepared. These are great points in favour of the malted milk, and those who have struggled with other milk preparations, including the more expensive and much advertised varieties, will readily appreciate their importance. The amount of barley malt in Horlick's is not sufficient to give the preparation that "beery flavour" to which some patients so strongly object; what little malt flavour remains is easily disguised by the addition of cinnamon. As a vehicle for administering such drugs as veronal this preparation of milk is excellent; as a food it stands very high. For institutional use it is supplied in large jars, which will be found more economical than the smaller tins or bottles.

